# Predictors of dementia after first ischemic stroke

**DOI:** 10.1590/1980-57642021dn15-020009

**Published:** 2021

**Authors:** Wafik Mahmoud El-Sheik, Aktham Ismail El-Emam, Ahmed Abd El-Galil Abd El-Rahman, Gelan Mahmoud Salim

**Affiliations:** 1 Neuropsychiatry Department, Menoufia University ‒ Shibin El-Kom, Egypt.; 2 Banha Mental Health Hospital ‒ Banha, Egypt.

**Keywords:** dementia, stroke, strategic infarction, demência, acidente vascular cerebral, infarto estratégico

## Abstract

Various mechanisms contribute to dementia after first ischemic stroke as lesions on strategic areas of cognition and stroke premorbidity.

**Objectives:**

Assessing clinical and neuroimaging predictors of dementia after first ischemic stroke and its relation to stroke location, subtypes and severity.

**Methods:**

Eighty first ischemic stroke patients were included. Forty patients with dementia after first stroke and forty patients without dementia according to DSM-IV diagnostic criteria of vascular dementia. All patients were subjected to general and neurological assessment, National Institute Health Stroke Scale (NIHSS) for stroke severity, Montreal Cognitive Assessment (MoCA) scale for cognition assessment, MRI brain and Trial of Org 10172 in acute stroke treatment (TOAST) classification for stroke subtypes.

**Results:**

Left hemispheric ischemic stroke, strategic infarctions, diabetes mellitus and stroke of anterior circulation were found to be independent risk factors for dementia after first ischemic stroke (OR=3.09, 95%CI 1.67-10.3, OR=2.33, 95%CI 1.87-8.77, OR=1.88, 95%CI 1.44-4.55**,** OR=1.86, 95%CI 1.45-6.54, respectively). Hypertension, dyslipidemia, smoking, ischemic heart disease, high NIHSS score and large vessel infarction were significantly higher among post stroke dementia patients. However, on binary logistic regression, they did not reach to be independent risk factors.

**Conclusion:**

Stroke location (left stroke, strategic infarction, anterior circulation stroke) and diabetes mellitus could be predictors of dementia after first ischemic stroke, but stroke severity, stroke subtypes, hypertension, dyslipidemia, smoking and ischemic heart could not.

## RESUMO.

Vários mecanismos contribuem para a demência após o primeiro acidente vascular cerebral (AVC) isquêmico como lesões em áreas estratégicas da cognição e morbidades pré-AVC.

**Objetivos:**

Avaliar os preditores clínicos e de neuroimagem de demência após o primeiro AVC isquêmico e sua relação com a localização, subtipos e gravidade do AVC.

**Métodos:**

Oitenta pacientes com primeiro AVC isquêmico foram incluídos. Quarenta pacientes com demência após o primeiro AVC e quarenta pacientes sem demência de acordo com os critérios diagnósticos do DSM-IV para demência vascular. Todos os pacientes foram submetidos à avaliação geral e neurológica, o *National Institute Health Stroke Scale* (NIHSS) para gravidade de AVC, a escala de Avaliação Cognitiva de Montreal (*Montreal Cognitive Assessment -* MoCA) para avaliação de cognição, ressonância magnética cerebral e classificação pelo *Trial of Org 10172 in acute stroke treatment* (TOAST) para subtipos de AVC.

**Resultados:**

AVC isquêmico do hemisfério esquerdo, infartos estratégicos, diabetes mellitus e AVC da circulação anterior foram considerados fatores de risco independentes para demência após o primeiro AVC isquêmico. (OR=3,09, IC95% 1,67-10,3, OR=2,33, IC95% 1,87-8,77, OR=1,88, IC95% 1,44-4,55, OR=1,86, IC95% 1,45-6,54, respectivamente). Hipertensão, dislipidemia, tabagismo, cardiopatia isquêmica, escore NIHSS alto e infarto de grandes vasos foram significativamente altos entre pacientes com demência pós-AVC. No entanto, na regressão logística binária, não chegaram a ser fatores de risco independentes.

**Conclusão:**

A localização do AVC (AVC esquerdo, infarto estratégico, AVC da circulação anterior) e diabetes mellitus podem ser preditores de demência após o primeiro AVC isquêmico, mas a gravidade do AVC, subtipos de AVC, hipertensão, dislipidemia, tabagismo e coração isquêmico podem não ser de risco.

## INTRODUCTION

Post-stroke dementia (PSD) is a common cause of dementia, with a prevalence ranging from 6 to 32%.^1^ It is a syndrome of multiple cognitive domains deficits including memory, may occur within three months after clinical stroke, affects daily functioning, quality of life, and ability to return to work, and increases the mortality rate after stroke by three times.^2^ Dementia and stroke relationship remains complex since several synergistic or cumulative mechanisms are involved as strategic vascular brain lesions, metabolic or vascular risk factors, previous silent infarcts, leukoaraiosis, accelerated pre-existing degenerative lesions, coincidence of Alzheimer's pathology, endothelial dysfunction, blood-brain barrier impairment, and neuroinflammation.^3^ Strategic infarctions are infarctions at areas related to cognition and behavior as frontal, temporal lobe, and hippocampus.^4^ Early detection of cognitive problems following stroke helps cognitive rehabilitation and decreases mortality.^5^

The primary objectives of this study were assessing the relation between dementia after first ischemic stroke and stroke location (strategic infarction, side of stroke), stroke subtype (anterior or posterior/large vessel or small vessel or cardioembolic), stroke severity and stroke risk factors. Secondary objectives involved evaluating which of the previous factors could be predictors of dementia after first ischemic stroke.

## METHODS

A cross sectional case control study was done in the period from March 2016 to March 2017. The authors obtained permission to conduct this study from the Research Ethics Committee of the Faculty of Medicine, Menoufia University. The study was performed according to principles of Helsinki Declaration. Eighty patients with ischemic stroke for the first time were recruited from Neurology department of Menoufia University Hospital. In this study, we excluded patients with evidence of chronic medical disease, other types of dementias, patients using drugs that can affect cognitive functions, patients with severe dysphasia, patients with history of cognitive impairment prior to stroke, and patients with hearing or visual impairment affecting ability to complete testing. All subjects underwent neurological history (stressing on risk factors of stroke as diabetes mellitus (DM), hypertension (HTN), smoking, dyslipidemia, ischemic heart disease (IHD) and atrial fibrillation (AF)) and examination expressing the initial stroke severity by National Institutes of Health Stroke Scale (NIHSS) which is composed of 11 items, with a score between 0 and 2 or 0 and 3 or 0 and 4 given to each item. Zero score is normal function, higher scores mean deficit. Minor stroke (1-4), moderate stroke (5-15), moderate to severe (16-20), and (21-42) severe stroke.^6^ Montreal Cognitive Assessment (MoCA) scale was done,^7^ taking 10 minutes to assesses global cognitive function and specific cognitive domains, which are visuo-spatial abilities, short-term memory, attention, concentration, working memory, language, abstract reasoning, orientation, and multiple aspects of executive functions. The total score is 30 points; a score of 26 or above is normal. Magnetic resonance imaging (MRI) of the brain was done, and we classified patients according to Trial of ORG 10172 in Acute Stroke Treatment (TOAST) classification^8^ for stroke subtypes into large artery atherosclerosis, cardioembolism, small-artery occlusion. We also classified ischemic areas into strategic areas of cognition and non-strategic ones, as well as affected areas into anterior and posterior circulation. Other lab tests were done, as kidney and liver function, lipid profile and thyroid function tests. From clinical and imaging, we classified patients into two groups according to Diagnostic and Statistical Manual of Mental Disorders - Fourth Edition (DSM-IV), a diagnostic criteria of vascular dementia:^9^ forty patients with PSD (PSD group) and forty without dementia (the control group). DSM-IV criteria include multiple cognitive deficits manifested by memory impairment plus one or more of the following: apraxia, agnosia and disturbance in executive functioning (i.e. planning, organizing, sequencing, abstracting). The cognitive deficits cause significant impairment in social or occupational functioning and represent a significant decline from a previous level of functioning. Focal neurological signs and symptoms (e.g., exaggeration of deep tendon reflexes, extensor plantar response) or laboratory evidence indicative of cerebrovascular disease are judged to be etiologically related to the disturbance. The deficits do not occur exclusively during the course of a delirium.

### Statistical analysis

The data were collected, tabulated, and analyzed by *Statistical Package for the Social Sciences* (SPSS) version 17.0 on IBM compatible computer (SPSS Inc., Chicago, IL, USA). Both descriptive and analytic statistics were used. Analytic statistics included the following tests: chi-square test, Fisher's exact test, Z test, t-test, Mann Whitney U test, and Binary logistic regression.

## RESULTS

**Figure 1.**
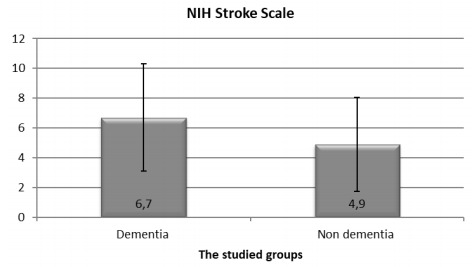
National Institute Health Stroke Scale among patients and controls.

PSD group and the control group (the non-demented) were matched regarding age and sex. The PSD group showed higher percentage of cases with DM, HTN, smoking, dyslipidemia and IHD, but there was no difference between the two groups regarding AF. NIHSS was significantly higher among PSD patients than the non-demented group. (Table 1 and Figure 1)

**Table 1.** Demographic and clinical characteristics of first stoke patients with and without dementia.

Sociodemographic	First ischemic stroke patients				Test	p-value
	I=dementia (n=40)		II=non-demented (n=40)			
Age/year X±SD	60.7±8.73		57.70±5.69		t-test	
Range	41-79		50-74		1.62	0.11
	No	%	No	%	chi-square test	
Sex Male	31	77.5	25	62.5	2.14	0.14
Female	9	22.5	15	37.5		
DM Positive	27	67.5	11	27.5	FE	<0.001
Negative	13	32.5	29	72.5	12.83	
HTN Positive	26	65.0	17	42.5	4.07	0.04
Negative	14	35.0	23	57.5		
Smoking Smoker	25	62.5	13	32.5		
Non-smoker	15	37.5	27	67.5	7.22	0.01
IHD Positive	15	37.5	3	7.5	10.32	0.001
Negative	25	62.5	37	92.5		
AF Positive	3	7.5	2	5.0	FE	1.0
Negative	37	92.5	38	95.0	0.21	
Lipid profile						
Normal	3	7.5	10	25.0	4.50	0.03
Dyslipidemia	37	92.5	30	75.0		S
NIH Stroke Scale						
X±SD	6.70±3.55	4.90±3.12	U test		0.02	
Range	2-18	0-12	2.42			

X: mean; SD: standard deviation; FE: Fisher's exact test; p-value<0.05=significant; NIH: National Institute Health.

**Figure 2.**
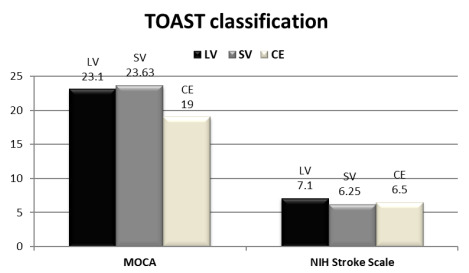
Relationship between Trial of ORG 10172 in Acute Stroke Treatment classification (atiology) and both Montreal Cognitive Assessment scale and National Institute Health Stroke Scale.

TOAST: Trial of ORG 10172 in Acute Stroke Treatment; MoCA: Montreal Cognitive Assessment; LV : large vessel; SV small vessel; CE: cardioembolic.

The PSD group had lower global cognitive function (lower total MoCA score) and lower scores in the following domains: executive function, naming, memory, language, and attention, but there are no significant differences regarding orientation and abstraction between the two groups. There was no significant relationship between TOAST classification and both MOCA scale and NIHSS (Table 2 and Figure 2).

**Table 2.** Montreal Cognitive Assessment of first stroke patients with post stroke dementia and these without post stroke dementia.

MoCA items	First stroke patients		Statistical analysis	
	Dementia n=40	Non-demented n=40	U	p-value
Executive functions X±SD	3.65±1.03	4.70±0.72		
Range	2-5	3-5	5.15	<0.001
Naming X±SD	1.80±0.76	2.9±0.30		
Range	1-3	2-3	6.33	<0.001
Memory X±SD	3.4±1.26	4.6±0.74		
Range	1-5	3-5	4.77	<0.001
Language X±SD	2.1±0.30	2.85±0.36		
Range	2-3	2-3	6.67	<0.001
Attention X±SD	4.60±0.87	5.55±0.60		
Range	3-6	4-6	4.84	<0.001
Abstraction X±SD	1.85±0.36	1.95±0.22		
Range	1-2	1-2	1.48	0.14
Orientation X±SD	5.45±1.13	5.75±0.54		
Range	2-6	4-6	0.77	0.44
Total MoCA X±SD	22.90±2.54	28.40±1.30		
Range	16-25	26-30	7.76	<0.001

MoCA: Montreal Cognitive Assessment; X: mean; SD: standard deviation; U: Mann Whitney U test; p<0.001; HS: highly significant; p>0.05=non-significant.

The PSD group showed more affection in left cerebral hemisphere and more strategic areas of infarction. Basal ganglia and frontal areas were the most common sites responsible for strategic infarct dementia in our study. There was significantly higher percentage of large vessel stroke in PSD group (45.0 *versus* 17.5%), while more patients with small vessel stroke were found in the non-demented group (77.5 *versus* 45.0%). The affected circulation was mainly the anterior circulation in PSD group (85 *versus* 55% in non-demented group) (Table 3).

**Table 3.** Site and cause of stroke (magnetic resonance imaging brain and TOAST classification).

MRI report	The studied groups				Test	p-value
	Dementia n=40		Non-demented n=40			
No	%	No	%	chi-square
Side of the lesion						
Right	14	35.0	23	57.5	4.07	0.04
Left	26	65.0	17	42.5		
Location of infarction					Z test	
Strategic areas	30	75	11	27.5	4.03	<0.001
-Thalamic -Basal ganglia -Angular gyrus -Hippocampus	10 8 3 9	25.0 20.0 7.5 22.5	4 2 1 4	10.0 5.0 2.5 10.0	1.47 1.69 0.51 1.21	0.07 0.04 0.30 0.11
Non-strategic areas	10	25.0	29	72.5	4.03	<0.001
Affected circulation						
Anterior	*34*	85.0	22	55.0	8.57	0.003
Posterior	*6*	15.0	18	45.0		
TOAST classification						
LV	18	45.0	7	17.5	8.96	0.01
SV	18	45.0	31	77.5		
CE	4	10.0	2	5.0		

TOAST: Trial of ORG 10172 in Acute Stroke Treatment; LV : large vessel; SV: small vessel; CE: cardioembolic.

On binary logistic regression for independent risk factors (predictors) of dementia after first ischemic stroke, left stroke, strategic infarctions, DM and stroke of anterior circulation were independent risk factors for dementia with odds ratio of 3.09, 2.33, 1.88 and 1.86 respectively, while stroke severity (NIHSS), large vessel stroke subtype, HTN, IHD, smoking, and dyslipidemia were not (Table 4).

**Table 4.** Binary logistic regression analysis for independent risk factors of dementia among stroke patients.

Risk factors	SE	Wald chi-square test	p-value	*Odds Ratio*	95%CI (lower‒upper)
DM	0.02	2.54	0.02	1.88	1.44-4.55
HTN	0.67	0.67	0.89	0.87	0.23-2.06
Smoking	1.02	1.43	0.13	1.12	0.65-3.44
IHD	0.33	0.97	0.34	0.95	0.12-2.33
Lipid profile	0.56	0.56	0.65	1.01	0.76-5.66
NIH Stroke Scale	2.30	1.26	0.17	1.21	0.99-3.45
TOAST classification	1.87	0.89	0.33	0.96	0.76-5.01
Left side of lesion	0.22	2.54	0.01	3.09	1.67-10.3
Strategic area affection	2.22	2.44	0.02	2.33	1.87-8.77
Anterior circulation affection	1.98	2.65	0.01	1.86	1.45-6.54

SE: standard error; 95%CI: 95% confidence interval; DM: diabetes mellitus; HTN: hypertension; IHD: ischemic heart disease; NIH: National Institute Health; TOAST: Trial of ORG 10172 in Acute Stroke Treatment.

## DISCUSSION

A lot of studies relate PSD to recurrence of stroke, but in our study we try to assess contributors to dementia after first ischemic stroke. Our study showed matching between PSD group and the control group (ischemic stroke without dementia) regarding age and sex. This goes with most of studies, although some ones showed that PSD group was more frequent in males due to the decreased adiponectin neuroprotective role with ageing in males, increasing risk of cardiovascular disease, HTN, atherosclerosis and DM.^10^ ^,^ ^11^

Regarding the relation between stroke risk factors and dementia after first ischemic stroke, there was positive association between dementia after first stroke and each of DM, HTN, dyslipidemia, IHD and smoking, but not with AF. Only from these association, DM reaches to be an independent risk factor for dementia on binary logistic regression, increasing the risk by 1.88 (OR=1.88, 95%CI 1.44-4.55). Concerning DM, our result goes with other studies who found association between DM and PSD.^12^ ^,^ ^13^ However, an earlier systematic review found no effect of DM treatment on cognitive decline.^14^ The role of DM in dementia after stroke could be explained by related glucose and insulin dysregulation, as insulin controls synaptic function and neurotransmitter receptors affecting on memory, increases axonal regeneration and neurite out growth in the brain,^15^ ^,^ ^16^ and by increased intracranial large or small vessel artery disease with DM, which increases blood viscosity and decreases cerebral autoregulation and blood flow.^12^ In respect of HTN, our result coincides with the results of Yamada et al.,^17^ but not with Tamam et al.^18^ study. A review showed that treatment of HTN especially by renin-angiotensin system (RAS) modulators helps in reduction of vascular dementia by 19-55% as they act on cognitive related brain regions.^19^ This is supported by episodic memory decline and hippocampal volume loss with RAS gene polymorphism.^20^ With regard to dyslipidemia, our result goes with Appleton et al.,^21^ and with Solomon et al.^22^ The present study found no difference between patients with PSD and patients without regarding AF, however other studies showed that AF was related to stroke, atrophy of hippocampus and impaired cognition.^23^ ^,^ ^24^ In our study, the demented group showed higher percentage of smokers, following the results of Kalaria et al.,^25^ who showed that cigarette smoking may contribute to cognitive impairment by increasing stroke risk, oxidative stress, and inflammation; but other studies, like Kao et al., are against this.^21^

With reference to the relation between stroke severity and dementia after first stroke, NIHSS score was higher in the PSD group, but on binary logistic regression stroke severity did not be a predictor for dementia. Surawan et al.^12^ found that stroke severity was associated with increased PSD risk. In this study, according to MoCA score, PSD patients showed lower following cognitive domains scores: executive function, naming, memory, language, attention. PSD occurrence and the cognitive domains affected may vary according to type, size, site and severity of stroke. For example, executive dysfunction is more with dominant prefrontal subcortical circuits' lesions.^20^ Regarding the relation between stroke site and dementia, high frequency of left cerebral ischemia (65%) was a predictor for dementia after first ischemic stroke as it increased by 3.09 folds with left sided ischemia (OR=3.09, 95%CI 1.67-10.3). This is in accordance with the results of Arauz et al., ^26^ and Renjen et al., ^27^ who found that vascular dementia risk increases with left-sided ischemic lesions by 5 folds (OR=5.0, 95%CI 1.92-14.1). Also Munsch et al.,^28^ has assessed cognitive outcome 3 months after stroke and reported that among independent predictors was left hemispheric stroke explaining the intimate relation between complex cognitive function and language, which is supported by a recent study.^29^ Also, strategic infarcts were predictors for dementia after first ischemic stroke as it increased by 2.3 folds with strategic infarction (OR=2.33, 95%CI 1.87-8.77). Basal ganglia and frontal areas were the most common sites responsible for strategic infarct dementia in our study. These results may be explained by disruption of frontal-basal ganglia-thalamus-cortical net as mentioned in Lanna et al. study^30^ and they are supported by Zhao et al.^29^ study that confirms the relation between stroke location and post stroke cognitive impairment, providing a map of strategic brain lesions involved in post stroke cognitive impairment specifying left angular gyrus, left caudate and palladium, left hemispheric tracts and the corpus callosum. Despite various studies confirm our result,^21^ ^,^ ^23^ ^,^ ^31^ ^,^ ^32^ ^,^ ^33^ ^,^ ^34^ Renjen et al. did not find a statically significant difference between strategic and non-strategic infarction as risk factor of post stroke cognitive impairment (8 *versus* 73%).^27^

About the relation between stroke subtypes and dementia, in this study, there was significantly higher percentage of large vessel ischemic stroke patients in PSD group in comparison to the non-demented group (45.0 *versus* 17.5%), while more patients with small vessel stroke were found in the non-demented group (77.5 *versus* 45.0%). However, large vessel stroke did not reach to be a predictor for dementia in our sample and we could not say that stroke subtype is an independent predictor for dementia after fist stroke. Our result is in harmony with Selnes and Vinters^35^ who stated that cognitive impairment is more in large infarctions than in small ones, unless the small infarction is in strategic area, as well with other studies which showed that large-vessel stroke with secondary PSD may result from relatively unusual (strategic) strokes involving branches of the main cerebral arteries.^25^ ^,^ ^36^ Our result does not go with Blanco-Rojas et al.^37^ nor with Grau-Olivares and Arboix^38^ who stated that more than half of cases of first lacunar stroke present with cognitive impairment and small vessel cerebral ischemia should be considered a prodrome of vascular subcortical dementia.

In our study, the affected circulation was mainly the anterior circulation in PSD group (85%) in comparison to only 55% in non-demented group. This difference reach to be predictor of dementia after first stroke increasing the risk by 1.86 fold (OR=1.86, 95%CI 1.45-6.54). Our result agrees with Chen et al.^39^ who stated that patients with anterior circulation infarction have more severe cognitive impairment and partially with Tu et al.^40^ who studied various types of cerebral infarction and prevalence of cognitive impairment and found lower Mini Mental Scale scores with partial anterior cerebral infarction (PACI), but the difference did not reach statistical significant level among the 3 types of cerebral infarction. Mellon et al.,^2^ however, found that MoCA scale was more impaired in posterior circulation (OR=1.86, 95%CI 1.84-1.89) after adjusting for age and stroke severity.

Our study has some limitations as the relatively small number of cases especially those with strategic areas affection which interfered with the assessment of how each of these areas affects global and specific cognitive domains. Patients were not assessed for depressive or psychotic symptoms. Cognitive state was assessed prior to stroke only by history. Finally, antihypertensive drugs were not adjusted among patients and controls. Future research is needed on a large group of patients for long period of follow-up, comparing dementia predictors of ischemic with hemorrhagic strokes, comparing first stroke with recurrent one, comparing cortical with subcortical lesions, using more advanced neuroimaging in precise localizing of strategic networks in the brain and for better understanding cognitive disorders in stroke.

Stroke location (left stroke, strategic infarction, and anterior circulation stroke) and DM could be predictors of dementia after first ischemic stroke. In spite of association between dementia after first stroke and stroke severity, large vessel stroke, hypertension, dyslipidemia, smoking, ischemic heart, they did not reach to be independent risk factors of dementia after first stroke.
